# From the last 100 to the first 100–outcome after a manufacturer change in reverse fracture arthroplasty

**DOI:** 10.1007/s00068-024-02724-3

**Published:** 2025-01-20

**Authors:** Johannes Gleich, Evi Fleischhacker, Christopher Lampert, Georg Siebenbürger, Ben Ockert, Wolfgang Böcker, Tobias Helfen

**Affiliations:** Department of Orthopaedics and Trauma Surgery, Musculoskeletal University Center Munich (MUM) University Hospital, LMU Munich, Marchioninistr. 15, 81377 Munich, Germany

**Keywords:** Reverse fracture arthroplasty, Elderly, Ortho-geriatric, Manufacturer

## Abstract

**Purpose:**

If surgery is indicated for elderly patients suffering a proximal humerus fracture, reverse fracture arthroplasty became the preferred type of treatment due to its good and reliable outcomes over the last decade. Surgeons could choose from a wide range of implants and up to now there was no evaluation, if a change of the manufacturer affects patients` outcome.

**Methods:**

The last 100 patients before and the first 100 after manufacturer change in reverse fracture arthroplasty were evaluated at a level one trauma center, all treated by only 3 senior shoulder surgeons. Clinical as well as radiographic outcome parameters were assessed, perioperative up to 24 months after surgery.

**Results:**

Mean age in both groups was nearly 80 years with comparable distribution of gender and comorbidities. A trend to shorter duration of surgery was observed after the change, mainly according to an uncemented fixation of the stem. During follow-up no significant differences, beneficial as well as negative, could be observed regarding clinical and radiographic outcome.

**Conclusion:**

A manufacturer change on the fly is possible without negative consequences for patients` outcome. Expertise of the whole OR-team as well as standardized training with the new implant seems to be a more important factor than a specific type of implant.

## Introduction

Reverse total shoulder arthroplasty covers an increasing number of pathologies at the glenohumeral joint. Immense expertise in primary (e.g. rotator cuff arthropathy) as well as in secondary indications (e.g. acute proximal humerus fracture and sequelae) was gained during the last decades, so today a shoulder surgeon could recommend this specific type of treatment with good evidence. Especially reverse fracture arthroplasty became the preferred type of treatment for irreparable proximal humerus fractures in the elderly due to its reliable outcomes.

Nevertheless, complications and subsequent revisions have to be considered and are evaluated in ongoing studies. Some complications were noticed as implant-associated during and after implantation, like insertion difficulties, dislocation, periprosthetic fracture or instability. Somerson et al. reported that implant-associated complications also vary between different manufacturers [1].

Besides choosing a specific manufacturer and type of implant, it was shown that outcome after surgery is affected by surgeons` expertise as well. Improvement in functional outcomes and a decrease of variability were already observed after increase in surgical expertise [2] [3]. The primary objective in surgical use of a reverse shoulder arthroplasty therefore should be: best time, best team, best implant. Different countries vary in compilation of this team.

In Germany, in the past musculoskeletal pathologies were treated separately, either by orthopedic surgeons or trauma surgeons. A merged education, a combined syllabus and common hospital structures were implemented since 2005. Besides a lot of medical and economical chances, as well as synergies from orthopedics and trauma surgeons, also challenges and changes take place up to now [4]. Subsequently, more and more departments joined forces and built common departments for orthopedic and trauma surgery.

At the study center, this unification was performed 2 years ago. As a consequence for a common department in shoulder and elbow surgery, a change of the manufacturer of reverse shoulder arthroplasty implants was resolved to standardize treatment. The present study should evaluate, if this change affected patients outcome after surgery as well as surgical performance of the specialists.

## Methods

Patients were prospectively as well as retrospectively included in this ethical board approved study (LMU No.: 156–12), which was conducted at a level one trauma center with two hospital sites. Only 3 senior shoulder surgeons (board certified orthopedic and trauma surgeons performing > 50 shoulder surgeries per year, including arthroscopic procedures, fracture treatment around the shoulder and shoulder arthroplasty), performed surgery.

Change of manufacturer was implemented on 01/08/2021 and without any transition period, from there on only implants from the new manufacturer were used. Before implementation, a standardized one-day training course was held in the new manufacturer's wet lab, where specific implant characteristics were reviewed on site and standardized implantation was practiced Previous Implant (Group A): Aequalis Reversed FX; Tornier, Wright Medical Group, Memphis, TN, USA (stem cementation is mandatory for this implant) New Implant (Group B): Univers Revers™ Shoulder System, Arthrex Inc., Naples, FL, USA (with this implant, stem cementation is optional).

The first 100 patients after and the last 100 patients before the change, suffering a 3- or 4-part-fracture (according to Neer-classification) of the humeral head, primary treated with a reverse fracture arthroplasty, were included. For accurate diagnostics, conventional radiography as well as computed tomography was used in all patients. Primary reverse fracture arthroplasty was indicated in patients ≥ 65 years, presenting with a dislocated 3- or 4-part- fracture with (partial) head-split or with a dislocated 3- or 4-part- fracture with intraoperative diagnosis of a rotator cuff tear ≥ grade II after Bateman. When a lesion of the axillary nerve, an open or pathologic fracture, insufficiency of the deltoid muscle or an infection of the extremity was seen preoperatively, patients were excluded.

All included patients were treated within 8 days of trauma by implantation of a primary reverse total shoulder arthroplasty. The procedure was performed in a beach chair position on a radiolucent table via a standardized deltopectoral approach. All Tornier stems had an inclination angle of 155° and were placed in 20° retroversion. All Arthrex stems were placed in an inclination angle of 135° and in 20° retroversion. Tuberosity fixation was performed in the Boileau technique with sutures and loops (Nice Loops; Tornier, Wright Medical Group, Memphis, TN, USA) or with with 1.7 mm SutureTapes (Arthrex Inc., Naples, FL, USA) according to manufacturers instructions. All patients received an abduction orthosis in the operations room (Type: SAS multi comfort (15° abduction); Medi, Bayreuth, Germany). The rehabilitation protocol allowed passive exercises on day 1 after surgery under supervision of a physiotherapist and unrestricted active range of motion after the third week.

For clinical follow-up examination, the Constant Score (CS), measurement of strength with a digital spring balance (Burg Wächter 76,000 Tara PS®), the range of motion (ROM) with a goniometer and the pain with a visual analog scale (VAS) were assessed [5]. External rotation was measured in axial plane with the arm by the patients’ side. The standardized follow-up included examination of the affected shoulder 6 weeks, 3, 12 and 24 months after surgery and at final follow-up.

In all patients true a.p., outlet view and axial radiographs were assessed the day after surgery and at every follow-up. Radiographs were evaluated for radiographic signs of tuberosity integration, resorption and displacement. Unless the tuberosities were dislocated, they were visible laterally on the stem and no more than 5 mm below the prosthetic head in diaphyseal continuity. When comparing the postoperative images and the radiographs at the follow-up visits, tubercular resorption was divided into less or more than 50%. Furthermore, scapular notching and loosening were assessed in this study. Data on length of stay, ASA-Score, implants used, surgery reports, radiographs and staff surveys were assessed via clinical database.

Peri- and postoperative complications were assessed according to previous published results by Barco et al.: instability, infection, notching, loosening, nerve injury, acromial and scapular spine fractures, intra-operative fractures and component disengagement. The applicability of each implant / manufacturer by the OR nurse was assessed with a scale according to academic grading in the authors country, with a range from grade 1 (“very good applicability”) to 6 (“insufficient applicability”).

Statistical analysis was performed on the data at final follow-up. Continuous variables were described by means and standard deviation and were compared using Mann Whitney Test. Categorical variables were analyzed using Fisher’s Exact Test. The significance level for all tests was set at p < 0.05. Statistical analysis was performed using SPSS (IBM Corp. Released 2016. IBM SPSS Statistics for Windows, Version 24.0. Armonk, NY: IBM Corp.)

## Results

**Fig. 1:**
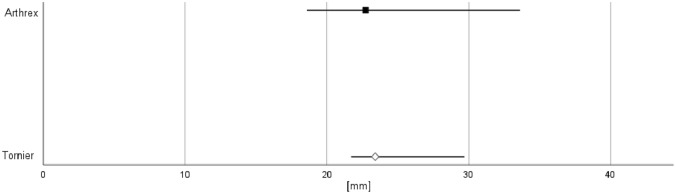
Distribution of the global lateral offset

**Fig. 2:**
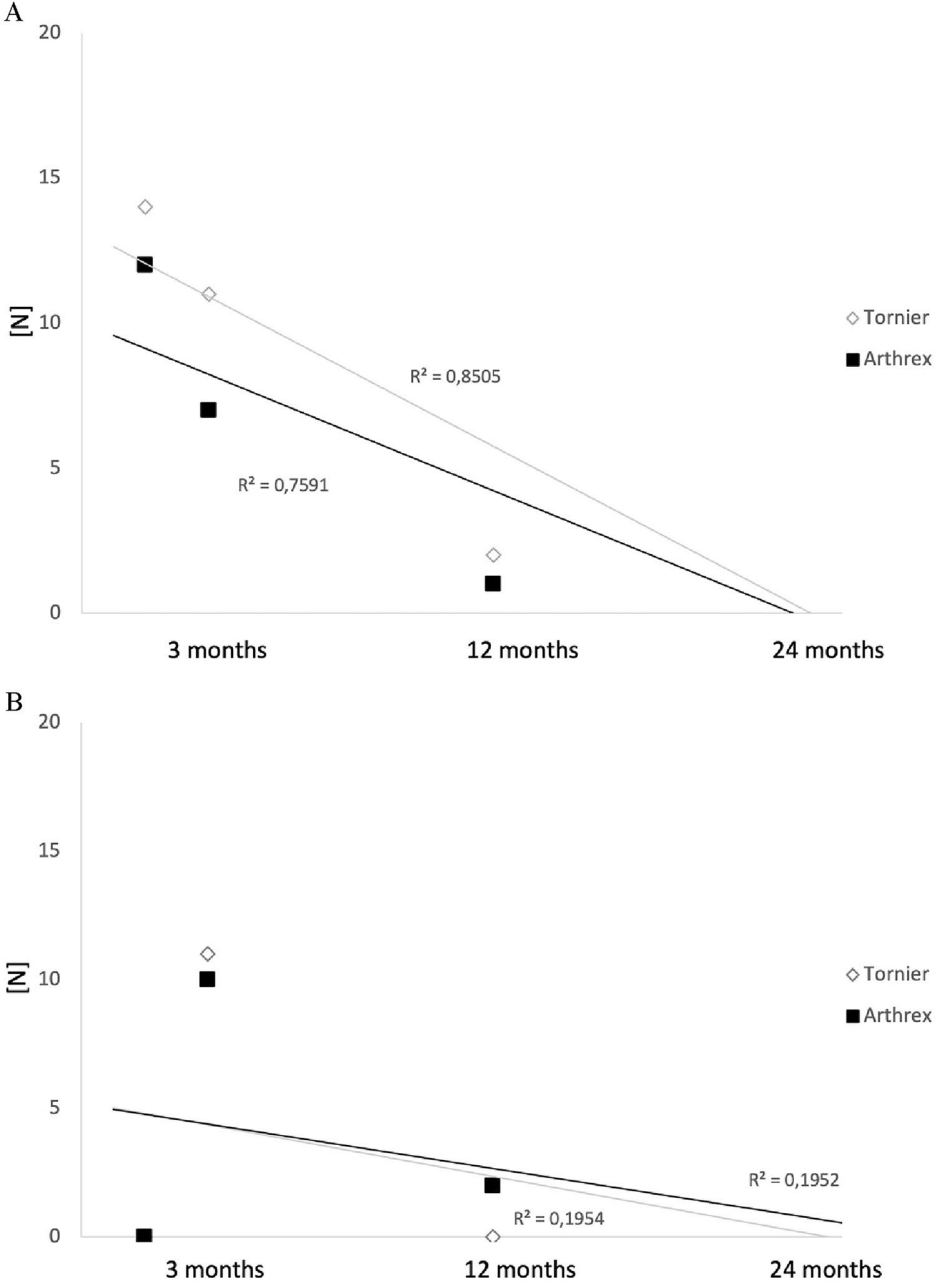
**A** Distribution of the greater tuberosity dislocation in the course of the follow-up. **B** Distribution of the greater tuberosity absorption in the course of the follow-up

**Fig. 3:**
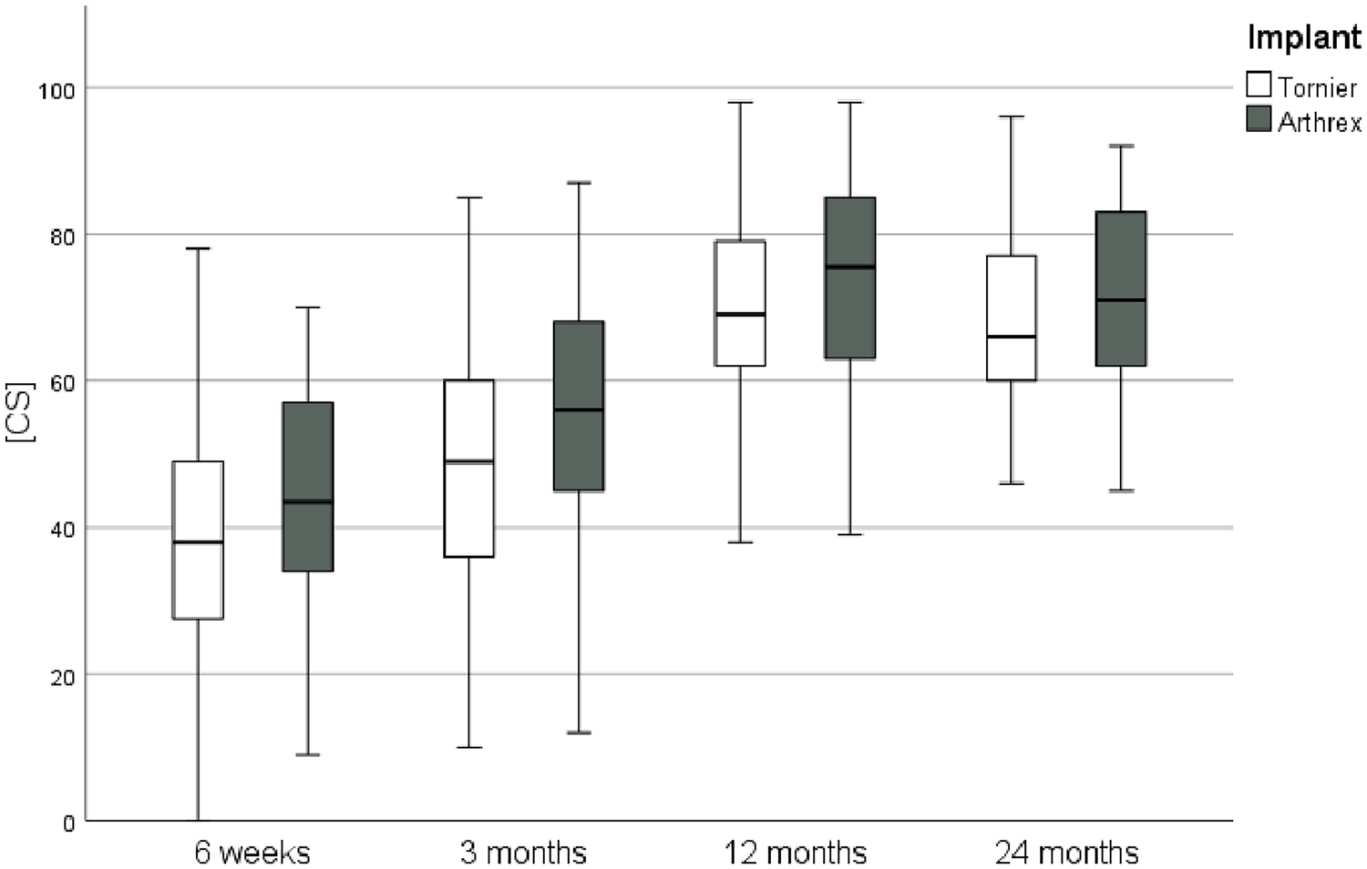
Constant Score during follow-up

Both groups showed an average age of nearly 80 years, most of the patients (> 80%) were female. There was no significant difference in comorbidities with an ASA-score of 2.6 ± 0.5, in time to surgery and hospitalization after surgery / period of intensive care unit. Same number of patients presented complaints on the affected shoulder joint before surgery as well as previous operations. A clear trend to a shorter duration of surgery was observed with 80.5 ± 26.9 min after manufacturer change, compared to 104.2 ± 24.3 min before. There was a significant reduction of stem cementation in group B (100 vs. 4, p = 0.01) and a slight, but statistically significant difference in global lateral offset (A: 23.4 ± 1.8, B: 22.8 ± 3.1, p = 0.03) (Table 1/Fig. 1). During follow-up, there was no significant difference in radiographic (dislocation / absorption of greater tuberosity, Fig. 2) as well as in clinical outcome parameters (Constant Score, infection) (Table 1/Fig. 3).

**Table 1:** Baseline data

	Tornier (A)	Arthrex (B)	[p]
Epidemiology			
Age [Years]	77.4 ± 9.1	79.8 ± 9.1	0.95
Sex [F/M]	83/17	81/19	0.71
ASA-Score	2.6 ± 0.5	2.6 ± 0.5	0.81
Trauma-surgery [days]	3.4 ± 4.3	3.4 ± 2.9	0.39
Total period of hospitalization [days]	9.9 ± 5.1	9.1 ± 4.2	0.2
Period of intensive care unit [days]	0.9 ± 2.4	0.6 ± 1.2	0.12
Anamnesis			
Complaints before surgery [N]	14	13	0.83
Previous operations [N]	3	3	1
Injuries after surgery [N]	0	0	1
Perioperative			
Incision-suture time [min]	104.2 ± 24.3	80.5 ± 26.9	0.75
Stem cementation [N]	100	4	**0.01**
Implant mismatch [N]	0	0	1
Iatrogenic fracture humeral [N]	0	3	0.08
Iatrogenic fracture glenoid [N]	0	0	1
Neurovascular complications [N]	0	0	1
Applicability by the OR nurse [Grade 1–6]	2 ± 0.8	2 ± 1	0.65
Preoperative reverse shoulder arthroplasty angle (RSA) [˚]	22.8 ± 6.4	23 ± 6.2	0.84
Postoperative reverse shoulder arthroplasty angle (RSA) [˚]	10.5 ± 5.1	11 ± 5.7	0.16
Global lateral offset [mm]	23.4 ± 1.8	22.8 ± 3.1	**0.03**
6 weeks follow-up			
*Drop-out [%]*	*1*	*0*	
Infection [N]	0	0	1
Joint dislocation [N]	0	0	1
Dislocation greater tuberosity [N]	14	12	0.83
Absorption greater tuberosity [N]	0	0	1
Fracture acromion [N]	0	0	1
Loosening stem [N]	0	1	0.31
Constant Score (CS) [pt]	39 ± 17.2	44 ± 14.5	0.18
3 month follow-up			
* Drop-out [%]*	*2*	*2*	
Infection [N]	0	0	1
Joint dislocation [N]	1	2	0.56
Dislocation greater tuberosity [N]	11	7	0.46
Absorption greater tuberosity [N]	11	10	0.88
Fracture acromion [N]	0	0	1
Loosening stem [N]	0	0	1
Constant Score (CS) [pt]	47.6 ± 16.4	55 ± 16.6	0.95
12 month follow-up			
*Drop-out [%]*	*7*	*2*	
Infection [N]	2	1	0.56
Joint dislocation [N]	1	1	1
Dislocation greater tuberosity [N]	2	1	0.56
Absorption greater tuberosity [N]	0	0	1
Fracture acromion [N]	0	0	1
Loosening stem [N]	0	0	1
Scapular notching [6]	51	42	0.2
Constant Score (CS) [pt]	70.8 ± 13.6	72.8 ± 14	0.48
24 month follow-up			
*Drop-out [%]*	*85*	*75*	
Infection [N]	0	0	1
Joint dislocation [N]	0	0	1
Dislocation greater tuberosity [N]	0	0	1
Absorption greater tuberosity [N]	0	0	1
Fracture acromion [N]	0	0	1
Loosening stem [N]	0	0	1
Constant Score (CS) [pt]	68.7 ± 12.7	71.8 ± 14.7	0.25

Parameters were bold reflected to higlight significance

## Discussion

Aim of the present study was to evaluate the influence of a manufacturer change in reverse fracture arthroplasty on patients` outcome as well as in the user collective of shoulder surgeons.

Despite including patients 65 years and older, a mean age of nearly 80 years was observed in both groups. This represents the current demographic trend of an aging society and suggests an age- and gender related affection of the outcome. Stenquist et al. found no difference in complications or functional outcomes between a younger (mean age 64 years) and older (mean age 78 years) collective of patients after reverse fracture arthroplasty, which underlines comparability [7].

The total length of inhospital-stay in both groups was not significantly different (9 vs. 10 days). These findings differ from results by Menendez et al., who stated a mean length of stay from 4 days after surgery for proximal humerus fracture (internal fixation or arthroplasty), but are consistent to studies from the authors country, where 11.5 and 14.6 days were noted after reverse fracture arthroplasty [8], [9]. Patient (age, comorbidities) and system (availability of surgical capacity, lack of postoperative ambulatory care) related factors may delay surgery and timing of discharge, so these differences could be attributed to variances in treated patients and treating countries.

No significant difference, but a clear trend in duration of surgery with decreased time (mean 104 vs 80 min) after change of manufacturer was observed. Other data on reverse fracture arthroplasty show a mean duration from 130 to 140 min. Our findings might be mainly attributed to cementless fixation of the stem after manufacturer change. Only in 4 cases of group B, there was a cemented fixation of the stem, which was caused by an insufficient metaphyseal anchorage or fracture in the calcar region. A reduced duration of surgery is already known with reduced occurrence of postoperative complications from other surgical procedures in the elderly and could be regarded as an unintentional benefit of manufacturer change [10], [11].

Various parameters were implemented in reverse shoulder arthroplasty to objectively compare baseline situation and postoperative results. One of them, the so called reverse shoulder arthroplasty angle (RSA), evaluates on preoperative radiographs the inclination of the inferior glenoid cavity. On postoperative radiographs, the filling of this gap is measured, which stands for correct baseplate inclination. An adequate correction of glenoid inclination is vital in correct implant positioning, as superior inclination is a risk factor for reduced range of motion, loosening, and instability [12]. Also functional outcome is influenced by the RSA, as patients with a postoperative RSA of 0–10° tended to better functional results [13]. To achieve neutral inclination of the baseplate, the preoperative RSA needs to be corrected.

With a mean preoperative RSA of 23° ± 6° in both groups (22.8° ± 6.4° vs. 23° ± 6.2°), our results are comparable to Boileau et al., who found a mean RSA of 25° ± 8 [14]. Postoperative RSA is also comparable to current literature in both groups with a mean of 11° ± 5° (10.5° ± 5.1° vs. 11° ± 5.7°). Uçan et al. found a mean RSA of 5.5 ± 10.1° in plain x-rays and of 10.4 ± 10.3° in CT-scans after surgery [13].

During follow-up, functional outcome showed no clinically relevant difference between the groups, both short and mid-term. Tuphe et al. report comparable results in an even longer follow-up period depending on the implementation of an early rehabilitation protocol [15].

Regarding overall complications as mentioned before, we observed no difference with changing manufacturers and found a comparable spectrum as stated in current literature [16].

Nevertheless, a detailed review of each specific complication is essential:

According to a review by Contreras et al. the incidence of periprosthetic infection after reverse shoulder arthroplasty ranges from 3 to 4%, although rates as low as 0.5% and as high as 6.7% have been reported [17]. Our study showed rates from 1% (Arthrex) to 2% (Tornier) and consequently a low overall periprosthetic infection rate regardless of the manufacturer.

The different application options of the stems are certainly the biggest difference between the two implants. Therefore, a close look at potential effects is necessary. In the present collective cementation changed from a necessity to a rescue option. At the same time, however, non-cementation may also harbor risks. Despite a case of stem loosening, the option of non-cementation is a big step forward, starting with the operation time and ending with better revisability. Our findings match with a systematic review by Phadnis et al. and their conclusion, that uncemented stems have at least equivalent clinical and radiographic outcomes compared with cemented stems when used for reverse total shoulder arthroplasty. They also report intra-operative humeral fractures (cemented: 0.5%, uncemented 1.2%) and stem loosening in several studies, so regardless of the implant, its fixation and surgical experience, this seems to occur in low quantity. With an intra-operative humeral fracture rate of 3% after change of manufacturer, also a learning curve should be considered in our collective. During implantation of the stem and inlay, supporting and neutralizing the elbow joint could reduce intra-operative fracture risk [18]. The authors also strongly recommend dosed impaction of the cementless stem and dosed insertion of the inlay. In addition, an intraoperative X-ray check for an iatrogenic fracture of the calcar is recommended. In doubt, cementation should be generously indicated.

Scapular notching, once a mainly radiologic finding, gained clinically relevant importance with growing long-term data. The reported incidence varies widely, ranging from 4.6% to 96%, with decreasing rates in recent studies. It is more common in the 135° than in the 155° stems [19]. Our data showed a trend to less notching in 135° prosthesis, but without significant difference between the groups. Correct implantation technique (lateral offset with inferior overhang of the glenosphere) therefore is crucial in avoiding scapular notching, despite profound knowledge of the specific implant and manufacturer. As there are various options for lateralization (lateralize on the humeral, on the glenoid or on both sides), some authors aimed for a better definition and measured the global lateral offset, summing up both factors. They found high implant-depending variability among different manufacturers [20]. This is also proven by our data, that show significant difference in global lateral offset between the groups.

Another parameter, that primarily was noticed on radiographic follow-up and became outcome relevant with increasing data, is the tuberosity union rate. Patients with healed tuberosities after RSA in proximal humerus fractures showed improved range of motion and satisfaction [21]. After manufacturer change, a growing trend to tuberosity union was observed with 62% in the Tornier and 70% in the Arthrex group. In detail, we found higher tuberosity dislocation (27% Tornier, 20% Arthrex) than resorption (11% Tornier, 10% Arthrex) without significance.

**Fig. 4:**
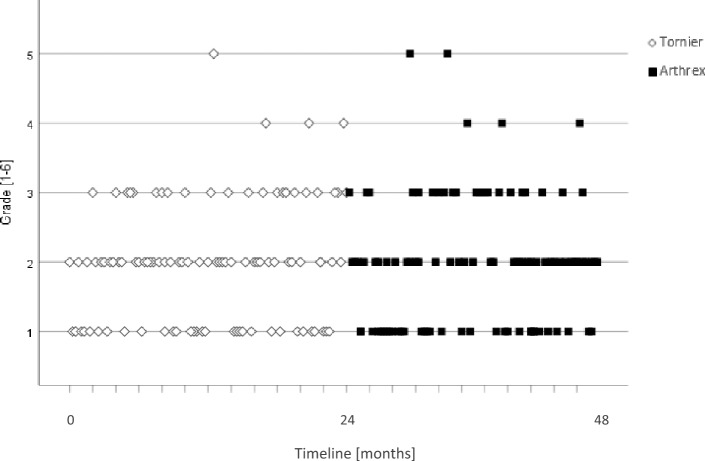
Applicability by the OR nurse [Grade 1–6]

Apart from all radiographic parameters, human factors are crucial for correct use of each implant and have a big impact on the outcome. Considering the team working together in the operation room, the scrub nurse as well as the surgeon have to know specific features of a new implant and how to use them. Nyberg et al. evaluated the experiences of operating room nurses and they stated to help solve problems as they arise, where there are obvious risks for patient complications [22] (Fig. 4).

## Conclusion

Concluding all clinical and radiographic findings, a change of the manufacturer in reverse fracture arthroplasty had no impact on patients` outcome in short as well in long-term. No negative effects, regarding length of hospital stay, perioperative complications or comprised clinical outcome during follow-up were observed, also no significant beneficial impact, despite a trend to a shorter duration of surgery due to uncemented fixation of the stem. The human factor, starting with the OR-staff and ending with the surgeon’s anticipation of possible pearls and pitfalls of an implant, seems to remain the most important parameter when introducing a new implant or manufacturer.

## Data Availability

The data that support the findings of this study are not openly available due to reasons of sensitivity and are available from the corresponding author upon reasonable request.
